# Internet addiction in young adults: The role of impulsivity and codependency

**DOI:** 10.3389/fpsyt.2022.893861

**Published:** 2022-09-06

**Authors:** Pierluigi Diotaiuti, Stefania Mancone, Stefano Corrado, Alfredo De Risio, Elisa Cavicchiolo, Laura Girelli, Andrea Chirico

**Affiliations:** ^1^Department of Human Sciences, Society and Health, University of Cassino and Southern Lazio, Cassino, Italy; ^2^Department of Human Studies, Communication, Education, and Psychology, Libera Università Maria SS. Assunta (LUMSA), Rome, Italy; ^3^Department of Human, Philosophical and Educational Sciences, University of Salerno, Fisciano, Italy; ^4^Department of Psychology of Development and Socialization Processes, Sapienza University of Rome, Rome, Italy

**Keywords:** Internet addiction, young adults, impulsivity, motor impulsivity, attentional impulsivity, codependency

## Abstract

Excessive Internet use has demonstrated comorbidity with other psychological symptoms and psychiatric disorders, as well as impairments in the management of daily life, relationships and emotional stability. Recent findings in the literature have consistently supported the relationship between impulsivity and Internet addiction. The present study hypothesized that, in addition to impulsivity, a further predictor of Internet addiction might be relational co-dependency, which is also associated in the literature with addiction phenomena, but mainly substance addiction. This paper investigates the role and predictive weight of impulsivity and codependency on Internet addiction on a sample of young adult university students (n = 481) by using a hierarchical regression analysis. The participants were administered the UADI-2, the BIS-11 and the SFCDS. In terms of percentage distribution, 38 % of the participants were in the dependency range, while 37.7 % demonstrated Internet abuse behavior. The results confirmed the role of impulsiveness (β = 0.312) and added to the literature by showing the significant role of relational codependency (β = 0.275), gender (β = 0.174) and age (β = 0.196). Thus, male participants were more dependent, more impulsive and more co-dependent, with increasing age in the given range (18–30). The present study shed light to the presence of this issue among young adults and that, as a preventive and restraining measure, there is a need not only for targeted awareness-raising programmes but also for interventions to promote greater emotional control and a more balanced management of personal relationships.

## Introduction

The Internet is one of the most widespread and accessible media for young people: chatting, role-playing, etc., are increasingly the routinary activities for them and the growing use of this media has led to the emergence of psychological problems linked to its possible maladaptive use in young people. The phenomenon of Internet abuse has been called by different names such as computer addiction, compulsive Internet use, Internet mania, problematic or pathological Internet use, and finally Internet Addiction (IA) (1–5). Young (6), Young and Rogers (1) bring Internet Addiction Disorder to the center of the scientific debate, shifting the diagnostic reference from substance-related problems to those found in pathological gambling problems (GAP) and in fact placing Internet addiction within impulse control disorders. Individuals with Internet addiction may lose control over their Internet use, resulting in impairments in the management of daily life, relationships and emotional stability (1, 2, 4, 7).

A critical level is identified when the excessive Internet use impedes the management of the young individual's developmental activities and negative consequences come to light in an overt way (for example, decline in school performance, excessive limitation of outside activities, permanent conflicts with parents and friends, etc.) (8–11). When it happens, except the use of Internet, several other activities and interests are neglected, despite they are consciously perceived as significant, while individual continue to massively use the Internet despite the possible harmful consequences, a phenomenon known as “harmful consumption” (12, 13).

Compared with the past, currently Internet abuse is classified not as an impulse control disorder ma as a (potential) addiction, i.e., the fact the tendency is to define addiction to specific online activities (as seen in section III of DSM-5 and ICD-11), rather than Internet addiction in general.

Currently, the main forms of addiction associated with the excessive use of Internet are: *Cyber-relational addiction*, characterized by an excessive tendency to establish friendship or love relationships with people met online, mainly *via* chat rooms, forums or social networks (14). In this condition, online relationships quickly become over-involving and individuals tend to neglect their relationships in presence with friends and family. *Information overload*, characterized by an obsessive search for information on the web: individuals spend increasing amounts of time searching for and organizing data on the web (15). *Cybersexual addiction*, which is characterized by compulsive use of pornography and virtual sex sites. Individuals usually download and use online pornography, engage in adult-only chats and may have compulsive masturbation (16). *Offline gaming*, characterized by a tendency to over-involve in virtual games that do not involve multi-player interaction and are not played over a network (17). *Online gaming*, in which excessive involvement and compulsive behaviors related to various online activities such as gambling, compulsive shopping, role-playing games are evident (18, 19).

Excessive Internet use has been found to be in co-morbidity with other psychological symptoms and psychiatric disorders (4). Internet addiction has been found to be associated with attention deficit hyperactivity disorder (20, 21), low self-esteem (22), shyness (23), depressive symptoms (1, 23–26), hostility (27, 28), interpersonal sensitivity (27, 29), disturbances in relationships (30, 31), obsessive-compulsive symptoms (OCS) (20, 24, 25), and impulsivity (32, 33).

Harmful Internet use, like substance abuse, triggers individuals' preoccupation with details, nervousness, irritability, aggression and impulsivity (4, 34). Previous studies have also shown that obsessive-compulsive symptoms are associated with the severity of Internet addiction (20, 24, 25). Cao et al. (32) reported that adolescents with Internet addiction show increased impulsivity and have various comorbid psychiatric disorders, which may be associated with Internet addiction. For those with behavioral inhibition issues, the Internet can serve as an area where individuals can receive short-term rewards through gaming, surfing or social networking, and be reinforced by immediate gratification (7, 35). A further study suggested that impulsivity can be considered as an endophenotype of addictive behavior (36). Impulsive individuals have problems in managing their behavior, showing recurrent failures to resist impulses to engage in a specified behavior and a feeling of lack of control while engaging in the behavior. A large body of the literature in this area concerns impulsiveness impacting the addictive tendencies (37, 38). Consistent with this, recent findings in the literature have consistently supported the relationship between impulsivity and Internet Addiction (33, 39–44).

Another construct that has been associated with addiction phenomena (predominantly substance addiction) is that of codependency. Codependency is often referred to as “relationship addiction”. It's an emotional and behavioral condition that interferes with an individual's ability to develop a healthy, mutually satisfying relationship. But over the years it's been expanded to include individuals who maintain one-sided, emotionally destructive, or abusive relationships (45–47). Researchers have identified several factors that are often linked with codependency: lack of trust in self or others; fear of being alone or abandoned; a need to control other people; chronic anger; frequent lying; poor communication skills; trouble making decisions; problems with intimacy; difficulty establishing boundaries; trouble adjusting to change; an extreme need for approval and recognition (48–50). The role of codependency among the variables associated with gambling disorder has been reported by Barrera-Algarín and Vázquez-Fernández (51). In contrast, an interesting contribution by Lu (52) recently illustrated the link between virtual community codependency and virtual community addiction: the virtual community codependency will need individuals to have a desire to derive compensation from the virtual community that cannot be achieved in the real world. If people in this community have similar needs, priorities, and goals, increasing the use of Facebook will lead to an increase in virtual community addiction. The author argues that codependency is a pattern of dysfunction in interpersonal relationships. According to the social compensation theory, if people feel insecurity and negative social identity in real life interpersonal networks, they may spend more time using virtual communities as compensation. Lu's study (52) tested and reported a direct impact of virtual community codependency on virtual community addiction. Furthermore, the increased use of Facebook when there is a sense of the ‘spirit of belonging together' can lead to increased tendency to virtual community addiction. In more general terms, Shishkov et al. (53) have first suggested a direct association between internet addiction and codependency, while, with reference to the set of patterns of thinking and behavioral characteristics of the codependent personality, Artemtseva and Malkina (54) pointed out that the codependents make cognitive errors about the consequences of their behavior in order to constantly protect themselves from uncertainty.

While the role of impulsivity has been widely analyzed in the literature of Internet Addiction, there is still a lack of studies that consider codependency as another possible factor associated to excessive Internet use. The present work had therefore the following objectives: evaluate the importance of Internet abuse and dependence in a sample of young adults, by also considering the gender of the participants; investigate the possible role of Impulsivity and codependency in explaining Internet Addiction. Other studies have confirmed for this age group the relationship between impulsivity and problems associated with various forms of addiction (55–59), and this can be even more true considering the important personal limitations in terms of mobility and relationships related to COVID-19 pandemic, which have not only solicited an increase in addictive practices (60, 61) but also a deterioration in perceived safety in relationships with others, amplifying the compensatory search for codependent relationship patterns that Internet use can offer (62–65). On the basis of the literature presented hitherto, we hypothesized that relational codependency might be in young adults, in addition to impulsivity, a further significant predictor of Internet addiction.

## Methods and materials

### Participants

Participants were recruited by forwarding an email to students enrolled at a university in central-southern Italy. This email defined the goals as well as the function of the study. Subjects were invited to enter a specific link found in the same notice, after which they filled in and posted the answers telematically and digitally. Participants were assured anonymity and also the use of information in aggregate type for research purposes. They also provided their written informed consent to participate in this study. The protocol was approved by the local university Institutional Review Board and tools administration took place in April and May 2020. A total of 1,500 emails were sent out. As far as the drop-out ratio is concerned, 86 participants dropped out after beginning to fill it in, therefore 481, including 219 (45.5 %) males and 262 females (54.5 %) with an average age of 21.79 and SD = 4.16 and age range 18–30, completed questionnaires were finally collected.

### Tools

- *Uso-Abuso e Dipendenza da Internet* [Internet use-abuse and addiction] (UADI-2), (66), assesses the psychopathological risk of Internet abuse and the psychological use that users make of the network (example items: “I happen to have flashbacks or disconnected thoughts during or after a long Internet connection”; “Sometimes I like to lie on the net”; “On the Internet I happen to look for erotic material or talk about sex”). The instrument measures the psychological and psychopathological aspects related to the use and abuse of the Internet and has been designed to be administered both off-line (by filling in the U.A.D.I. in paper form) and on-line (by filling it in *via* Internet). The instrument consists of 24 items that the person must answer on a 5-point scale ranging from 1 (Absolutely false for me) to 5 (Absolutely true for me). The UADI-2 allows scoring with reference to four dimensions: Dissociation (describes some dissociative symptoms as bizarre sensory experiences, de-personalization, de-realization, along with the tendency to alienation and estrangement-escape from reality), Impact on Real Life (contains items describing the real-life consequences i.e., any changes in habits, social relationships, mood as a result of continued Internet use), Addiction Symptoms (contains items that address some behaviors and symptoms of addiction, particularly with reference to gradually increasing linkage period, abstinence, compulsiveness, and hyperinvolvement), Identity and Sexuality (contains items describing manipulation of true personal identity online and the tendency to search for sexually oriented content). The scoring has three score ranges: up to 62, normal Internet use; 63–74, Internet abuse; over 74, Internet addiction. Cronbach's alpha for this study was 0.867.
- *Barratt Impulsiveness Scale-11 [BIS-11*; (67, 68)] is a 30-item self-report questionnaire designed to assess general impulsivity taking into account the multifactorial nature of the construct. The structure of the instrument allows the assessment of six first-order factors (attention, motor, self-control, cognitive complexity, perseverance, cognitive instability) and three second-order factors: attentional impulsivity, motor impulsivity (motor and perseverance), unplanned impulsivity (self-control and cognitive complexity). Example items: “I do things without thinking”; “I act on the spur of the moment”; “I often have extraneous thoughts when thinking”. The person is asked to respond regarding how often he or she generally (not referring to a specific time interval) acts and thinks similarly to the items on the scale. The total score is obtained by summing up the first and second order factors. The items are distributed on a four-point scale (Rarely/Never = 1, Occasionally = 2, Often = 3, Almost Always/Ever = 4). In the present study, the Italian version by Fossati et al. (68) was used. Cronbach's alpha for this study was 0.835.
- *Spann-Fisher Codependency Scale* [SFCDS; (69)]. Codependency is referred as a dysfunctional pattern of relating to others with an extreme focus outside of oneself, lack of expression of feelings, and personal meaning derived from relationships with others. The tool is an unidimensional 16-item 6-point scale, ranging in score from 16 to 96 with higher scores reflecting codependency (example items: “It is hard for me to make decisions”, “I don't usually let others see the “real” me”, or “When someone upsets me I will hold it in for a long time, but once in a while I explode”). The mean Spann-Fischer co-dependency score is approximated with a midpoint of 52.6, a “high” score of 67.2 and a “low” score of 37.3 suggested by Fischer, Spann, and Crawford (69). The codependent person puts a lot of effort into satisfying the needs of others, constantly trying to be helpful and organizing others' lives, losing sight of and disregarding their own needs. For the purposes of this study, we obtained an Italian version of the questionnaire through back-translation procedures. We performed an exploratory factor analysis (Maximum Likelihood, promax rotation) on The Italian Spann-Fischer Codependency Scale items. Our results revealed a one-dimensional structure. A test for internal consistency and item-total correlations confirmed that excluding one poor functioning item, best preserved the reliability of the questionnaire, and we therefore decided to exclude it from the final Italian version. After this adjustment, the scale consisted of 15 items and showed good internal consistency (Cronbach's α = 0.820).

### Statistical analysis

Descriptive analyses (percentages, means, standard deviation, skewness and kurtosis, confidence intervals); *t*-test for comparison of scores with respect to gender; Pearson's bivariate correlations; testing of univariate and multivariate regression assumptions; and hierarchical regression were conducted.

## Results

Descriptively, 38.0% (*n* = 183) of the sample were in the range of Internet addiction (with a mean score on the UADI-2 > 74). The 27.7% (*n* = 133) of the sample were found to be in the Internet abuse range (with a mean score between 63 and 74). The remaining 34.3% (*n* = 175) were in the normal range of Internet use. Significant differences emerged, however, in relation to gender. Amongst males, 45.2% (*n* = 99) were addicted to the Internet, while 30.1% (*n* = 66) had Internet abuse behavior. Among females, 32.1% (*n* = 84) were addicted, while 25.6% (*n* = 67) abused the Internet. These differences were more specifically highlighted in Table 1 where the *t*-test comparisons between the two groups and the respective breakdowns in the range of full dependency, abuse and normal Internet use are shown.

**Table 1:** Differences in the level of Internet addiction with respect to gender of participants.

	**Males**	**Females**			
Dependence	M (SD)	M (SD)	CI 95%	*p*	d
	86.703 (8.37)	82.71 (4.95)	[1.26; 5.37]	* < 0.005*	0.48
Abuse	M (SD)	M (SD)	CI 95%	*p*	d
	67.79 (3.42)	68.78 (3.46)	[−2.17;0.192]	*>0.05*	0.28
Normal use	M (SD)	M (SD)	CI 95%	*p*	d
	55.48 (6.14)	53.42 (5.93)	[0.092; 4.02]	<0.05	0.34

In Table 2 below it can be seen that the level of male dependence was higher both in terms of the overall score and in relation to the subscales of Dissociation, Identity and Sexuality and Impact on Real Life, while the manifestation of Addiction Symptoms did not significantly differ between genders (*p* > 0.05).

**Table 2:** General and specific dimensions of Internet addiction with respect to gender of participants.

	**Males**	**Females**			
General addiction	M (SD)	M (SD)	CI 95%	*p*	d
	73.00 (14.33)	66.74 (13.57)	[3.75; 8.76]	* <0.001*	0.45
Dissociation	M (SD)	M (SD)	CI 95%	*p*	d
	21.21 (4.38)	19.16 (4.35)	[1.27; 2.84]	* <0.001*	0.46
Real life impact	M (SD)	M (SD)	CI 95%	*p*	d
	14.18 (2.40)	13.61 (2.46)	[.129; 1.00]	<0.05	0.24
Addiction symptoms	M (SD)	M (SD)	CI 95%	*p*	d
	23.05 (5.77)	22.54 (5.59)	[−0.515; 1.52]	>0.05	0.09
Identity and sexuality	M (SD)	M (SD)	CI 95%	*p*	d
	14.56 (5.10)	11.43 (5.11)	[2.21; 4.05]	<0.001	0.61

Table 3 below presents the descriptive statistics of all the variables used in the study.

**Table 3:** Descriptive statistics of the variables.

	**Skewness**	**(*SE*)**	**Kurtosis**	**(*SE*)**	**Mean**	**(*SD*)**
Age	0.757	0.111	−0.932	0.222	21.79	4.16
General Internet addiction	0.123	0.111	−0.560	0.222	69.59	14.26
Dissociation	−0.020	0.111	−0.411	0.222	20.09	4.48
Real life impact	−0.026	0.111	0.495	0.222	13.87	2.45
Addiction symptoms	−0.272	0.111	−0.362	0.222	22.77	5.67
Identity and sexuality	0.083	0.111	−0.891	0.222	12.85	5.33
Codependency	−0.275	0.111	0.495	0.222	51.36	10.88
Total impulsivity	−0.410	0.111	−0.242	0.222	68.03	10.47
Motor impulsivity	−0.150	0.111	−0.726	0.222	22.87	5.18
Attentional impulsivity	−0.183	0.111	−0.343	0.222	18.04	3.23
Non planning	−0.122	0.111	0.630	0.222	27.12	4.34

SE, Standard Error; SD, Standard Deviation.

Table 4 below shows the bivariate correlations between the measures used in the study. It can be seen that there were significant associations with both the Codependency scale (0.347^**^) and the Impulsivity scale (0.349^**^). More specifically for the latter measure, Internet Addiction reported correlations with the subscale of the Attentional Impulsiveness (0.379^**^) and Motor Impulsiveness (0.365^**^), while the association with the subscale of non-planning was not significant.

**Table 4:** Bivariate correlations.

	**1**	**2**	**3**	**4**	**5**	**6**	**7**	**8**	**9**	**10**
General Internet addiction (UADI-2)	1									
Dissociation (UADI-2)	0.861**	1								
Real life impact (UADI-2)	0.387**	0.288**	1							
Addiction symptoms (UADI-2)	0.832**	0.615**	0.038	1						
Identity and sexuality (UADI-2)	0.887**	0.676**	0.293**	0.628**	1					
Codependency (SFCDS)	0.347**	0.230**	0.017	0.394**	0.306**	1				
Total impulsivity (BIS-11)	0.349**	0.320**	0.248**	0.157**	0.382**	0.138**	1			
Motor impulsivity (BIS-11)	0.365**	0.335**	0.233**	0.190**	0.384**	0.168**	0.878**	1		
Attentional impulsivity (BIS-11)	0.379**	0.312**	0.198**	0.218**	0.428**	0.182**	0.805**	0.647**	1	
Non planning (BIS-11)	0.124**	0.140**	0.174**	−0.010	0.144**	−0.003	0.765**	0.443**	0.425**	1
Age	0.173**	0.092*	0.089*	0.136**	0.225**	0.232**	−0.110**	−0.077	−0.022	−0.169**

N = 481.

^**^Correlation is significant at the 0.01 level (2-tailed). ^*^Correlation is significant at the 0.05 level (2-tailed).

For Age Spearman's correlation has been used. Pearson's for the other variables.

In order to identify predictors of Internet addiction, a hierarchical regression was performed on the variables of Codependency and Impulsivity. The preliminary verifications of the regression assumptions excluded the presence of multivariate outliers. Mardia's multivariate kurtosis index (62.33) was in fact below the critical value [*p* (*p* + 2) = 99]; therefore, the relationship between the variables can be considered substantially linear. Low co-linearity was indicated by the low variance inflation factor (VIF) values <2 and high tolerance values > 0.60. For verification of the assumptions on the residuals, the average between the standardized and raw residuals was equal to 0; the Durbin–Watson test had a value of 1.96 and was therefore indicative of the absence of autocorrelation.

A hierarchical multiple regression was run to determine if the addition of Codependency, Impulsivity, Age, and Gender improved the prediction of the Internet Addiction. The full model resulted statistically significant, *R*^2^ = 0.289, F(4,480) = 48.119, *p* < 0.001; adjusted *R*^2^ = 0.283.The regression model included Codependency and Impulsivity at step 1, Age at step 2, Gender at step 3. The results of the hierarchical multiple linear regressions are presented in Table 5. In the regression model, with Internet Addiction as outcome variable, Codependency and Impulsivity jointly explained a 22% portion of the outcome variability. Adding Age at the second step provided a significant improvement in the explained variance, which reached 26%. By adding Gender at the third step, the explained variance further significantly increased to 29%. Standardized beta values were significant. with a positive sign for Codependency, Impulsivity, Age, and a negative sign for Gender. The order reflects the relative importance assigned to each predictor. Since this study intended to give special emphasis as a predictor to codependency, agreeing with what has been argued in this regard in the recent literature cited above, this variable appears to have taken precedence in the entry over that of impulsivity, which is dominant in the less recent literature. As a third consideration, age was included, with respect to which some studies reported an inverse association with the level of addiction (70–72), while others reiterated the linear direction with increasing levels of Internet addiction (73–75). It was interesting to understand what the predictive relationship between age and problematic internet use might be in the sample of young adults considered. Finally, the gender variable was included, which according to other studies is predictive of different male and female susceptibility to problematic and pathological internet use. Thus, it was deemed that the four variables, considered in this order of entry into the predictive model, could provide a significant explanatory portion of the phenomenon under study.

**Table 5:** Results of hierarchical linear regression analyses.

	**Outcome variable**		
	**Internet addiction**		
**Independent variable**	**Adjust R^2^**	**ΔR^2^**	**β**
*Step 1*	0.220***	0.223***	
Codependency			0.310***
Impulsivity			0.318***
*Step 2*	0.255***	0.037***	
Codependency			0.265***
Impulsivity			0.342***
Age			0.197***
*Step 3*	0.283***	0.029***	
Codependency			0.275***
Impulsivity			0.312***
Age			0.196***
Gender			−0.174***

N = 481; β = standardized beta value.

***p ≤ 0.001.

## Discussion

The present study was aimed to evaluate the importance of Internet abuse and dependence in a sample of young adults and it aimed to clarify the possible role of impulsivity, codependency, gender and age in explaining Internet addiction. Among the instruments in the Italian context to measure Internet addiction, the *UADI*, although not recent, has been preferred over others such as the *Generalized Problematic Internet Use Scale-2* [GPIUS-2, (76); Italian valid. (77)] or the classic *Internet Addiction Test* [IAT, (1); Italian valid (78)], because, in addition to having in other studies confirmed good psychometric properties (79–83), it allowed us to assess two dimensions not present in the other instruments mentioned above, and which we considered significant for their possible association with the impulsivity and codependency variables, namely dissociation experiences and identity manipulations on the web. First of all, the results showed a substantial percentage of young people in the addiction phase (one third of the total sample). Moreover, another third of the sample demonstrated Internet abuse behavior. This clearly indicates that there was an issue of control over the use of the Internet among the young adults involved. Nevertheless, we recognize that there might be an overestimation, especially referred to the classification of “abuse” of the Internet. This can be due to the fact that the instrument was originally carried out in 2005 when the average use of the Internet and social networks was still limited. Over the years, we have seen a significant increase in the use of the Internet, especially among young people, due to a natural expansion of connectivity possibilities and as a normal evolution of a behavior of consultation and search for information. Moreover, the use of messaging for interactions with friends and acquaintances has also highly increased. Another aspect that should definitely be considered is that the UADI does not differentiate between different forms of addiction (smartphone, social media, cybersex, game addiction), while it measures a general prevalence of addiction. In light of current developments, we believe there is a need to provide adequate distinctions between different types of addiction and to differentiate areas affected by possible problems. Considering that the administrations took place after the period of greatest impact of the COVID-19 pandemic in Italy (84) which, as we know, imposed a prolonged isolation and reduction in direct contacts, it is probable that these percentages are affected by the impact of social isolation (85, 86) and that this has contributed to a compensatory search on the Internet. The results are, however, similar to the findings of the study by Salarvand et al. (87), also conducted with university students. Consulting the existing literature related to the period of COVID-19 lockdown (the same period in which we conducted our survey), has shown that the rates of general addiction increased as compared to the pre-COVID period. For example, the study of Burkauskas et al. (88) has shown that Internet Gaming Disorder (IGD) has increased 1.6 times (compared to the pre-COVID period) while the prevalence of the Problematic Internet USE (PIU) has increased 1.5 times. The same increase (1.6 times) during the COVID-19 pandemic of PIU has been also remarked by (89) in both adults and young people. This increase is particularly critical among young people as pointed out by several studies. For example, Zhao et al. (90) estimated the PIU prevalence rate in a sample of university students to be 28.4%, while a Swiss study by Mohler-Kuo et al. (91) estimated the PIU prevalence rate to be 21.3% for young adults.

Of particular interest, however, is the recent meta-analysis by Meng et al. (92), which includes 504 studies from 64 countries conducted before November 2021 and from which the importance of the varying incidence of specific modes of Internet addiction can be clearly understood. The study reports prevalence estimates of 26.99% (95% CI, 22.73–31.73) for smartphone addiction, 17.42% (95% CI, 12.42–23.89) for social media addiction, 14.22% (95% CI, 12.90–15.65) for Internet addiction, 8.23% (95% CI, 5.75–11.66) for cybersex addiction, and 6.04% (95% CI, 4.80–7.57) for game addiction.

Underlying the differences in prevalence estimates among the studies should certainly be noted the incidence of the instrument used. In our case, the results reported using the UADI-2 suffer from a lack of classificatory articulation and a normative update that may be reflected in some overestimation of problematic incidence.

However, in the enforced form of preventive isolation, a vicious circle is created that pushes people to seek comfort, entertainment, distraction and relief on the Internet, putting aside the real discomforts, which in this way are not resolved and addressed (93). In other words, the Internet acts as a deterrent and an escape route for people who experience difficulties in socializing in real life. Due to character traits such as shyness or situations of social isolation, the use of new technologies and social networks seem to become a privileged source of intense and satisfying emotions and sensations, albeit originating from entirely virtual dimensions, so that the Internet can represent a means of escaping from everyday reality and taking refuge in an illusory and gratifying world, in which the virtual element makes it possible to overcome the difficulties and inhibitions that can characterize real interactions, thus triggering pathological mechanisms that severely affect the social relationships, the financial situation and the mental health of the people involved (92).

Internet addictions are more frequent in people with a basic emotional fragility. They are triggered in people who are already experiencing psychological difficulties such as depression, obsessive-compulsive disorders and anxiety disorders (94). The immoderate and improper use of mobile phones and the Internet not only can cause huge gaps between people, but can also lead them to withdraw into themselves, to develop relational insecurities or a fear of rejection, to feel inadequate and in need of support, even if this is external and for its own sake. It should not be forgotten that among these forms of addiction, there is also the so-called ludopathy, i.e., addiction to games and gambling, to which mobile devices also contribute on a large scale (95, 96).

Our results underline the male prevalence of Internet addiction, in line with other studies carried out during the same period (97, 98). Regarding gender differences, the literature indicates that men are generally attracted to sex sites and online games. Women are more likely to spend time flirting in chat rooms. Men prefer visual stimuli and focused on sexual experiences, while women are more focused on relationships and interactions (99–102). These features are congruent with the findings regarding gender comparisons of the UADI-2 addiction scale components. The significantly higher score on the dissociation scale for males is associated with increased gaming [see also (103–105)], whereas the score on the identity and sexuality scale is more likely to relate to behavior related to searching the Internet for sexually oriented content or masking one's identity in chat rooms or role-playing games [see also (106, 107)]. While no gender differences were found with regard to the manifestation of specific addiction-related symptoms, the negative impact on real life (work, study, social relationships, general wellbeing) was greater for males.

The analysis of the bivariate correlations clearly confirmed both the association with impulsiveness and that with codependency. The subsequent hierarchical regression also confirmed the hypothesis of the present study. In terms of the weights of the regression coefficients, impulsivity remains the main predictor (β = 0.312), as indicated by most of the above literature, but it is flanked by co-dependency, which shows a regressive weight just below the former (β = 0.275).

To the best of our knowledge, the only study that explicitly relates codependency to Internet addiction is that of Shishkov et al. (53). Their contribution shows that higher levels of Internet addiction were associated with an increase in codependency. Although the authors do not carry out a regression analysis, but limit themselves to correlation associations, they comment on the results, pointing out that the prerequisites for Internet addiction as well as for codependency are in the family.

In contrast to the study of Shishkov et al., in which both Internet addiction and codependency were greater in younger individuals, our results show the opposite trend: within the 18–30 age group, it is the older participants who are more dependent, both on the Internet and in terms of relationships. This result is particularly relevant as it raises interesting questions about the potential extension of addiction problems into the fully adult age group.

Some confirmation with respect to the age trend involved in such issues comes from studies that have recently focused on the Internet addiction of workers and professionals (108–111). Other studies also point out the association between Internet addiction (in both adults and young adults) with depression (43, 112–114), hyperactivity and attention deficit (115–119).

The prevalence of Internet addiction in the adults leads us to consider the growing incidence of attention disorders such as ADHD in this age group. Although ADHD is a disorder that begins in childhood, if it is not recognized and properly treated, it can develop into adult ADHD. Although hyperactivity often tends to diminish over time, emotional restlessness and instability in interpersonal relations sometimes persist, together with difficulty in organizing oneself and managing several tasks in parallel (120–123); attention difficulties persist, manifesting themselves as difficulties in tasks such as keeping appointments and meeting deadlines. These consequences negatively affect different aspects of the adult's life, often leading to financial and work difficulties, interpersonal and relationship problems (124, 125). The significant association and predictive estimation, which emerged in our study, of motor and attentional impulsiveness with Internet addiction, suggests that at the basis of this addiction there may also be problems of attention and impulse management that can be traced back to adult ADHD.

As regards codependency, this predictor usually includes personal relationship problems, also within the family context. We found only one study that explicitly considered family functioning, attentional impulsivity and Internet addiction in a sample of young adults in a single explanatory model (43). In this model, attentional impulsivity is proposed as a mediator of the relationship between family functioning and Internet addiction. Although our study does not test this mediation, it has shed light to the role of these predictors in explaining Internet addiction.

### Practical implications of the study

Once some of the possible significant predictors have been identified, it seems appropriate to identify the containment interventions to be put in place. In this regard, the review by Xu et al. (126) on psychological interventions on Internet addiction suggests the formation of targeted and personalized intervention programmes. For impulsivity, which has been proposed as a potential indicator and treatment target of Internet addiction (127, 128), The Reality Therapy approach is suggested to assist individuals in controlling their behavior and making alternative Internet-related choices (129). Reality therapy is based on choice theory, which holds that people are in charge of their lives and what they do, feel, and think (126, 130). It focuses on goal-directed choices and self-control, which are very important aspects for young people (131, 132) directly by assisting individuals in reflecting on their behaviors, evaluating their options, and planning to choose more effective options (130, 133). Reality therapy may help people with addictions and impulsivity issues improve their self-control and reduce problem behaviors. Despite the fact that there have been very few studies of Internet addiction intervention using reality therapy alone, this method has been linked to improved self-esteem. Similar effects have been observed in studies of reality therapy for substance abuse (134, 135). Although more research is needed, preliminary findings suggest that reality therapy may play a role in the treatment of Internet addiction (130). Because good family functioning was linked to a lower risk of experiencing Internet addiction, family factors may be important targets for Internet addiction interventions (136). Family therapy is not a specific process, but rather a set of interventions aimed at improving family functions and relationships rather than directly addressing addictive behaviors. The therapies are designed to improve communication and relationships while shifting psychological needs fulfillment away from the internet and toward interactions and building relationships with family members (137, 138). Shek et al. (139, 140) used a combination of motivational interviewing and family-based therapy. Participants reported less Internet addiction and improved family functioning.

Since our study reveals the predictive role of codependency, and this is certainly associated with problems of poor relationship functioning, it can be assumed that both family therapy and other interventions or compound approaches may help. Mindfulness-oriented recovery enhancement (MORE), for example, combines mindfulness training with cognitive restructuring (the process of learning to identify and modify maladaptive thoughts through methods such as logical disputation) (141). Some studies have looked into combining two different psychosocial treatments. According to Yao et al. (142), combining reality therapy and mindfulness meditation had a significant effect on Internet gaming disorder.

Given that an inverse relationship between internet addiction and information literacy has emerged in several studies (143–145), further preventive and restraining interventions could include ad hoc media and information literacy enhancement programs, which have been found to be effective in addressing other youth issues such as various addictions (146–148), doping consumption in sports (149, 150), eating disorders (151–153), ciberbullismo (154, 155), youth aggressiveness and deviant behaviours (156, 157).

With regard to the above-mentioned interventions, it should be noted that since most of them are conducted with small groups of adolescents, it remains open to question the extent of their effectiveness with a different target group such as young adults and adults. For example, both adult co-dependency and adult hyperactivity problems would require further experimentation, taking into account the different contexts and the actual limitations/opportunities of the current living conditions. Further research and implementation of targeted and customized programmes will certainly be necessary.

### Limitations of the study

Our findings should be interpreted while acknowledging some limitations. First, the sample size for this study was small and the statistical power can be affected. This limitation was due to the difficulty of getting more students involved in the study during the COVID-19 emergency, but we believe that future studies could benefit from a larger sample size and selecting participants from other parts of the country. Second, the participants in our sample were all university students. This choice was made bearing in mind the results of recent meta-analyses conducted in different countries that have shown a high prevalence of Internet addiction in this population [e.g., (87, 158, 159)] and have raised the urgence to orientate policy strategies to this emerging issue for young adults. However future research will be needed to replicate these findings in other groups. Third, it should considered that the UADI-2 instrument does not differentiate between different forms of addiction (smartphone, social media, cybersex, game addiction) and the measure is indicative of a general prevalence, which in light of current developments, would instead need a specific distinction to adequately and differentially define the areas affected by possible problematicness. Furthermore, results reported may reflect some overestimation of problematic incidence due to this lack of classificatory articulation and normative update since the moment of validation of the instrument UADI-2 carried out in 2005. In addition, future studies could include more variables (such as socio-economic status, including clinical data as depression, anxiety, feeling of loneliness, interpersonal issues, maladaptive cognitions) and more covariates variables. Finally, it was a cross-sectional study, therefore, causalities could not be entirely clarified.

## Conclusion

This study investigates the role and predictive weight of impulsivity and codependency on Internet addiction on a sample of young adult university students by using a hierarchical regression analysis. The results confirmed that both impulsivity and codependency play a role in problems related to Internet use, moreover they showed the relative importance of gender and age. The study demonstrated that maladaptive and addicted use of the Internet is a critical issue also among young adults, and it suggests that preventive and restraint measures are needed. These can include not only targeted awareness programs, but also interventions aimed at encouraging a greater emotional and attentional control and a more balanced management of personal relationships among young people.

## Data availability statement

The raw data supporting the conclusions of this article will be made available by the authors, without undue reservation.

## Ethics statement

The studies involving human participants were reviewed and approved by Institutional Review Board of the University of Cassino and Southern Lazio. The participants provided their written informed consent to participate in this study.

## Author contributions

PD, SM, and SC designed the study and drafted the manuscript. PD, SM, SC, and ADR analyzed the data and discussed the results. EC, LG, and AC revised the manuscript. All authors contributed to the article and approved the submitted version.

## Conflict of interest

The authors declare that the research was conducted in the absence of any commercial or financial relationships that could be construed as a potential conflict of interest.

## Publisher's note

All claims expressed in this article are solely those of the authors and do not necessarily represent those of their affiliated organizations, or those of the publisher, the editors and the reviewers. Any product that may be evaluated in this article, or claim that may be made by its manufacturer, is not guaranteed or endorsed by the publisher.
